# Water Dynamics in Cancer Cells: Lessons from Quasielastic Neutron Scattering

**DOI:** 10.3390/medicina58050654

**Published:** 2022-05-12

**Authors:** Murillo L. Martins, Heloisa N. Bordallo, Eugene Mamontov

**Affiliations:** 1 Oak Ridge National Laboratory, Neutron Scattering Division, Oak Ridge, TN 37831, USA; 2 Niels Bohr Institute, University of Copenhagen, Universitetsparken 5, 2100 Copenhagen, Denmark; bordallo@nbi.ku.dk

**Keywords:** quasielastic neutron scattering, cancer cells, water dynamics

## Abstract

The severity of the cancer statistics around the globe and the complexity involving the behavior of cancer cells inevitably calls for contributions from multidisciplinary areas of research. As such, materials science became a powerful asset to support biological research in comprehending the macro and microscopic behavior of cancer cells and untangling factors that may contribute to their progression or remission. The contributions of cellular water dynamics in this process have always been debated and, in recent years, experimental works performed with Quasielastic neutron scattering (QENS) brought new perspectives to these discussions. In this review, we address these works and highlight the value of QENS in comprehending the role played by water molecules in tumor cells and their response to external agents, particularly chemotherapy drugs. In addition, this paper provides an overview of QENS intended for scientists with different backgrounds and comments on the possibilities to be explored with the next-generation spectrometers under construction.

## 1. Introduction

Cancer is one of the leading causes of death worldwide. In 2019, the last year in which the incidence and mortality rates of cancer were reported, it was among the two leading causes of death of individuals younger than 70 years old in 112 of 183 countries and ranked third or fourth in other 23 countries [1]. The increasing incidence and mortality of cancer are often (and reasonably) associated with the aging and growth of the population and with socioeconomic-related aspects, but controversially, cancer incidence in young adults is a rising concern in the medical community [1,2]. It is important to study how cancer cells respond to different external stimuli and environmental factors, their progression from benign to malignant cases, and the development of chemo- and radiotherapy resistances [3,4,5].

In the biology field, living cells and tissues, including those from cancer, are traditionally investigated with the main focus on morphological features (by using microscopy techniques), and on the behavior and response of the several cellular components as well as their cross-talk mechanisms. In the last few decades, this viewpoint has gained valuable support from materials science beyond microscopy, thanks to the development of experimental and computational analytical methods. For example, the mechanical properties of cancer cells are potentially associated with their migration and adhesion abilities [6], and dielectric signatures from different cancer cell lines have been already identified [7,8].

Within this context, the dynamics of water in cancer tissue environments has also been investigated, especially with experiments based on nuclear magnetic resonance [9,10]. Water is not only the main component of cells but also plays an indispensable role in the stabilization of DNA’s double helix, catalysis, protein dynamics and structure, cell migration, effects of ionizing radiation and chemotherapy drugs, and many other biological processes [11,12,13,14,15,16]. In addition, as already probed in protein solutions and even in living cancer cells, water is engaged in dynamics strongly coupled with the cellular biomolecules dynamics [17,18]. Hence, the dynamics of water molecules does reflect the global dynamics of the cellular components, even though these occur on different time and length scales.

Since materials science secured its spot as a powerful asset to support biological research, the neutron scattering community evolved towards multidisciplinary research. With this, various studies using neutron scattering have been performed to comprehend the dynamics of water in living cells, for example, even when not carried out on cancer cells per se, the data complement the investigations performed with nuclear magnetic resonance [19,20,21,22,23,24]. The latter, while powerful, is devoid of information on sub-micron and sub-nanosecond dynamics and does not provide a spatial characterization of the probed motions, which are not limitations of neutron scattering techniques.

In this review, we particularly address the works performed to this date with Quasielastic neutron scattering (QENS) on cancer cells and highlight the value of this technique in untangling the behavior of water molecules in tumors both pre and post the application of anticancer agents. This review paper is intended to provide an overview of the QENS technique and also describe the perspectives for future investigations based on the next-generation spectrometers under construction: MIRACLES, to be installed at the European Spallation Source in Lund, Sweden, and BWAVES, which will operate at the Spallation Neutron Source Second Target Station in Oak Ridge, TN, USA.

## 2. The QENS Technique: A Brief Overview

### 2.1. Basic Concepts of QENS

Quasielastic neutron scattering, QENS, is a technique that explores the unique interactions between neutrons and matter to probe microscopic dynamics in bulk and confined systems. Neutrons are non-charged subatomic particles found in all atomic nuclei (except for ^1^H) that have a comparable mass to protons, a magnetic moment of −1.913 μ_b_, and a nuclear spin of ½. Since neutrons exhibit a wave-mechanical duality, their momentum can be described either as $\vec{p}=m\vec{v}$, where *m* is the neutron mass (1.675 × 10^−27^ kg) and $\vec{v}$ its velocity, or $\vec{p}=\hbar \vec{k}$, where |$\vec{k}$| = (2π)/λ is the wave vector of the neutron and λ its wavelength. Hence, the neutron energy can be described as:

(1) $$E=\frac{p^{2}}{2m}=\frac{1}{2}mv^{2}=\frac{h^{2}}{2m\lambda^{2}}=\frac{\hbar^{2}k^{2}}{2m}$$

where $h=2\pi \hbar =6.626.10^{-34}$ J/Hz is the Planck’s constant.

Neutrons have different energies depending on the production sources and moderators. For QENS, the so-called “cold” neutrons are mostly used, and they have energies in the order of 20 meV (while 25 meV is an equivalent of 290 K) that are comparable to intermolecular energies and wavevectors on the order of molecular dimensions.

**Figure 1:**
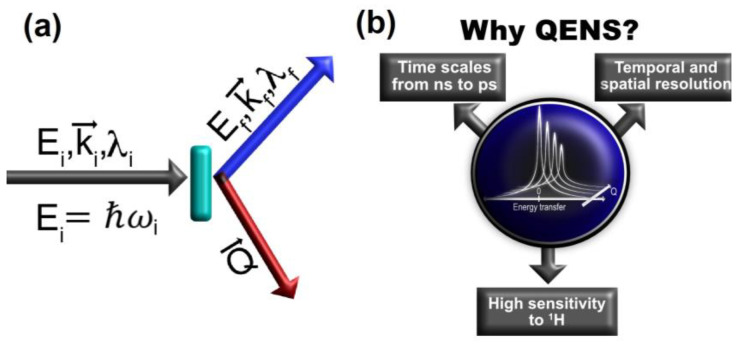
(**a**) Typical scattering triangle showing incoming neutrons with incident energy *E_i_*, wavelength *λ_i_*, and wave vector $\vec{k_{i}}$ interacting with the sample and assuming final energy *E_f_*, wavelength *λ_f_*, and wave vector $\vec{k_{f}}$. The momentum transfer $\vec{Q}$ is defined as the change in wave vector ($\vec{Q}$= $\vec{k_{i}}$− $\vec{k_{f}}$); (**b**) summary of the main features of the QENS technique. These are explained in detail in the text.

In a typical QENS experiment, the transfers of energy ($\Delta E=E_{i}-E_{f})$ and momentum ($\vec{Q}=\vec{k_{i}}-\vec{k_{f}},$ being $\left|\vec{Q}\right|=\frac{4\pi .sin\theta }{\lambda }$ if $\left|\vec{k_{i}}\right|=\left|\vec{k_{f}}\right|$ and 2θ is the scattering angle) between the incoming neutrons beam and the sample are evaluated as illustrated in Figure 1a. Therefore, fundamentally, QENS is an inelastic neutron scattering technique in which Δ*E* is small in comparison with the incident neutron energy. The combined evaluation of ΔE and $\vec{Q}$ makes QENS a unique technique for the characterization of relaxations within a variety of systems. While ΔE provides the time scale of such relaxations, since E=ℏω, their length scale is assessed by $\vec{Q}$, thus providing concomitant temporal and spatial probe capabilities.

The main features of QENS are summarized in Figure 1b. With this technique, one can probe dynamic events on the time scale of 10^−9^ s to 10^−12^ s, such as the translational and rotational motions of molecules, and obtain a spatial insight of such motions. In a comparison with other neutron scattering techniques that are commonly employed in biology-related research, the vibrational modes observed with Inelastic Neutron Scattering (INS) are on the time scale of 10^−14^ s to 10^−15^ s and mostly contribute to the background in a QENS experiment. In addition, crystallography experiments widely used to resolve the structure of drugs, proteins, and others rely on coherent scattering interactions between neutrons and matter where the Bragg reflections are important (the differences between coherent and incoherent scattering are explained further in the paper). The signals collected by detectors largely affected by Bragg reflections are commonly discarded for the QENS data analysis [25,26].

Neutrons, as non-charged particles, scarcely interact with the electrons cloud, which allows for great penetration into matter, especially compared to other scattering probes such as infrared. In biomedical-related research, this feature has been explored to investigate the dynamics of drug molecules confined in different polymeric/ceramic formulations, which is not readily possible with techniques based on light scattering [26,27,28,29]. Additionally, numerous possibilities for sample environments in experimental setups can be explored due to the high penetrative power of neutrons. In addition, QENS benefits from the exceptional sensitivity of neutron scattering to natural hydrogen atoms (^1^H). With this, the dynamics of ^1^H-rich species not only can be probed but also masked out during an experiment via selective isotopic substitution of ^1^H by deuterium (^2^H).

While planning neutron scattering experiments, one must always consider the nature of the interactions between neutrons and matter. These interactions are defined by the so-called scattering cross-section (*σ*), which describes the probability of neutrons being scattered by different isotopes. During an experiment, all neutrons with an energy $E_{f}=E_{i}-dE$ scattered into a certain solid angle dΩ are counted by the detectors, and the measured intensity is proportional to the double differential scattering cross-section given by:

(2) $$I\;\propto \frac{\partial^{2}\sigma }{\partial \Omega \delta E}={\left(\frac{\partial^{2}\sigma }{\partial \Omega \delta E}\right)}_{coh}+{\left(\frac{\partial^{2}\sigma }{\partial \Omega \delta E}\right)}_{incoh}$$

In Equation (2), the first term on the right side of the equation accounts for the coherent contribution, which reflects the collective motions of the atoms/molecules in a sample. In QENS experiments, particularly in the cases discussed in this review, the collective motions probed by the coherent part of the neutrons scattering are either very slow, as in correlated atomic motions, or very fast as in the cases of phonons vibrations, both of which minimally contribute to the detected signal. Coherent scattering is also most relevant in neutron diffraction experiments performed without measuring energy transfer and aimed at probing the sample structure. Likewise, coherent scattering gives rise to the signal representing large-scale structures in small-angle neutron scattering experiments.

**Figure 2:**
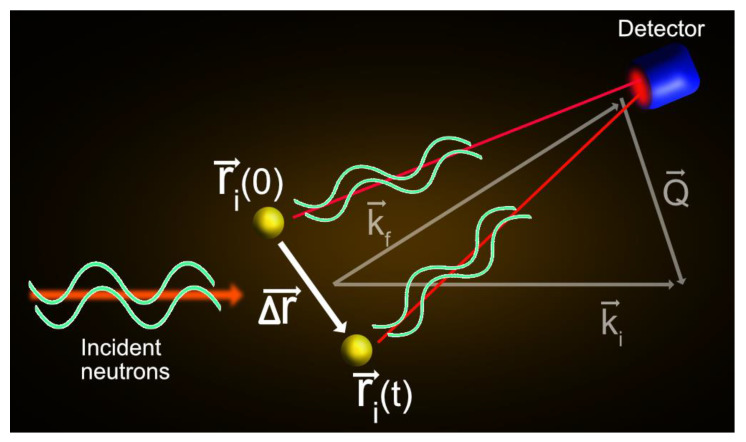
Graphical representation of a single particle scattering. The incoming neutron waves are scattered by a particle *i* at different times, 0 and t, and positions, $\vec{r_{i}\left(0\right)}$ and $\vec{r_{i}\left(t\right)}.$ The scattered waves interfere with each other before detection. The scattering triangle is also represented in the figure.

For QENS, the second term on the right side of Equation (2) is generally dominant and refers to the incoherent contribution. It accounts for neutrons scattered by the same particle at time 0 and at a given time *t* (single-particle dynamics) (see Figure 2). Hence, the incoherent scattering depicts the average single-particle dynamics ($△\vec{r}$) within the system, which can be conveniently associated with the trajectories calculated by molecular dynamics simulations [30]. Typically, there is a symbiotic relationship between QENS and molecular dynamics simulations, in which the latter can be used to interpret QENS results, and conversely, QENS can be used to benchmark and improve the potentials used in the simulations. This area of research has grown considerably in the last few years [31,32,33,34]. As well described by Zaccai [30], there is a reasonable perspective that this QENS/molecular dynamics interplay will soon be extended to the complex machinery of a living eukaryotic cell.

In practice, in neutron scattering experiments, a combination of the coherent and incoherent contributions is always measured. However, since hydrogen atoms have an exceptionally high incoherent scattering cross-section as compared with any other isotope, the coherent scattering contribution in the signal from ^1^H-rich samples is usually negligible except when strong structural peaks are encountered. In such cases, the detectors placed at scattering angles under strong influence from Bragg reflections are typically discarded, allowing for the single-particle dynamics from ^1^H-rich species to dominate the analysis. Therefore, from now on, we only focus on the incoherent part of the double differential scattering cross-section. As depicted in Equation (3), ${\left(\frac{\partial^{2}\sigma }{\partial \Omega \delta \omega }\right)}_{incoh}$ is related to the individual particle’s incoherent scattering cross-section, $\sigma_{incoh}$, and the incoherent dynamic structure factor, *S_incoh_*(*Q*, ω), as:

(3) $$\frac{\partial^{2}\sigma }{\partial \Omega \delta \omega }={\left(\frac{\partial^{2}\sigma }{\partial \Omega \delta \omega }\right)}_{incoh}=\frac{1}{4\pi N}\frac{\left|\vec{k_{f}}\right|}{\left|\vec{k_{i}}\right|}\left[\sigma_{incoh}S_{incoh}\left(Q,\omega \right)\right]$$

Here, *N* is the total number of scattering centers, $\left|\vec{k_{f}}\right|$ and $\left|\vec{k_{i}}\right|$ are the magnitudes of the final and initial wave vectors of the neutron, respectively, and $Q=\left|\vec{Q}\right|=\vec{|k_{i}}-\vec{k_{f}|}$. *S_incoh_*(*Q*, *ω*) contains the information on the single-particle dynamics within the sample and is a time-Fourier transformation of the so-called intermediate scattering function, *I_incoh_*(*Q*, *t*). Upon additional space-Fourier transformation, *I_incoh_*(*Q*, *t*) gives the single-particle self-correlation function, *G_self_*(*r*, *t*), which can be interpreted as the probability of finding a particle at position “*r*” at a time “*t*” if it has been at the position r = 0 at the time t = 0.

**Figure 3:**
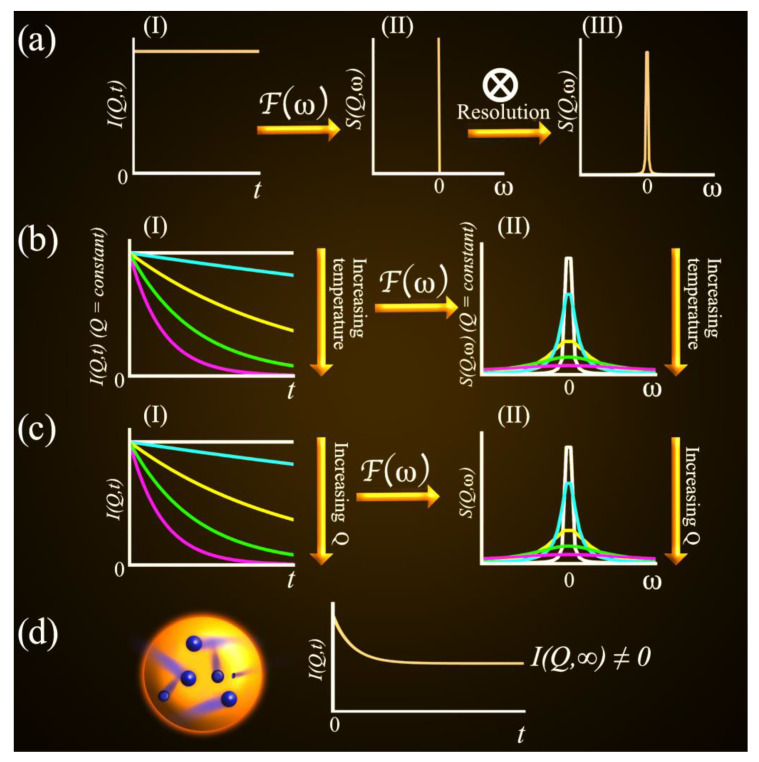
Schematics of the relationship between *I_incoh_*(*Q*, *t*) and *S_incoh_*(*Q*, ω). In (**a**), curve a-I shows the expected behavior of *I_incoh_*(*Q*, *t*) for a static particle where no exchange of energy occurs between the incoming neutrons and the sample. As shown in a-II, after a time-Fourier transformation of *I_incoh_*(*Q*, *t*), one obtains a δ-function-shaped *S_incoh_*(*Q*, ω), which broadens when convoluted with the experimental resolution (see a-III). In (**b**), the particles under analysis perform motions upon, for example, heating and *I_incoh_*(*Q*, *t*) gradually decays (b-II) and *S_incoh_*(*Q*, ω) becomes broader (b-II). In (**c**), an unconstrained diffusive motion is depicted, which leads to a gradual decay of *I_incoh_*(*Q*,*t*) over Q (c-I) reaching the limit of *I_incoh_*(*Q*, ∞) = 0, and *S_incoh_*(*Q*, *ω*) broadens as a function of *Q* (c-II). In (**d**), the motions of particles within a constrained geometry are depicted and *I_incoh_*(*Q*, *t*) plateaus at a finite value at t→ ∞.

Following the definitions above, schematics of the relationship between *I_incoh_*(*Q*, *t*) and *S_incoh_*(*Q*, *ω*) are presented in Figure 3. In Figure 3a, curve a-I shows the expected behavior of *I_incoh_*(*Q*, *t*) for a static particle. The probability of finding the particle over time is constant and equals one. In this case, no energy transfer between the incoming neutrons and the particle under analysis ought to occur, and only elastic events are perceived in the experiment. Following a time-Fourier transformation of *I_incoh_*(*Q*, *t*), one obtains *S_incoh_*(*Q*, *ω*) as a simple δ-function, as depicted in II in Figure 3a. However, in a real experiment, *S_incoh_*(*Q*, ω) is shaped by the inherent resolution of the spectrometers, which, as shown in III in Figure 3a, is convoluted to the collected data. The resolutions of QENS spectrometers depend on their design and are usually improved at the expense of other experimentally relevant factors, most notably, the measurement statistics. For example, the so-called high-resolution backscattering spectrometers have a narrower resolution function (thus allowing for assessing slower motions) than the time-of-flight spectrometers. However, this feature is achieved at the expense of the signal-to-noise ratio and the more limited range of accessible values of energy transfer. Followed with Figure 3b, as the particle under analysis starts to perform motions (by warming up the sample, for example), *I_incoh_*(*Q*, *t*) gradually decays, and, as long as such motions are faster than the constraints imposed by the instrumental resolution, they are perceived as additional broadenings in *S_incoh_*(*Q*, *ω*), II in Figure 3b.

With the schematics in Figure 3c,d, the reader can intuitively comprehend the evolution of the QENS signal as a function of *Q* depending on the nature of the motions performed by the particles under analysis. For an unconstrained long-range diffusive motion, *I_incoh_*(*Q*, *t*) rapidly decays as *Q* increases and reaches the limit of *I_incoh_*(*Q*, ∞) = 0, as shown in I in Figure 3c, and *S_incoh_*(*Q*, ω) broadens accordingly as depicted in II in Figure 3c. In such a scenario, in real-space and time domains, the probability of finding a freely diffusing particle vanishes at very long times, and such an effect is even more pronounced at short distances from the origin of the motion. For particles moving within a constrained geometry, such as a confined translation or rotation of a molecular group, *I_incoh_*(*Q*,*t*) plateaus at a finite value at t→∞ (that is *I_incoh_*(*Q*, ∞) ≠ 0) (I in Figure 3d). In these cases, the broadening of *S_incoh_*(*Q*, ω) assumes a Q-independent constant value (aside from experimental fluctuations) and, since *I_incoh_*(*Q*, ∞) ≠ 0, an elastic component, that is a time-independent component of *I_incoh_*(*Q*, t), is introduced in the QENS signal and has been defined as the Elastic Incoherent Structure Factor (EISF). The determination of the time-independent component in *I_incoh_*(*Q*, t) is limited by the instrumental resolution at long times. For less constrained geometries, it is sometimes possible to observe the broadening of *S_incoh_*(*Q*, *ω*) assuming a Q-independent behavior at low Q-values and then evolving to a Q-dependent behavior at higher Q [35,36].

### 2.2. QENS Data Analysis

In most QENS experiments, the measured scattering intensity can be analyzed having the following empirical expression as a starting point:

(4) $$S\left(Q,\omega \right)=\left[x\left(Q\right)\delta \left(\omega \right)+\left(1-x\left(Q\right)\right)S_{incoh}\left(Q,\omega \right)\right]⊗R\left(Q,\omega \right)+B\left(Q,\omega \right)$$

In Equation (4), *x*(Q) refers to the fraction of particles that are either immobile or slower than the time scale defined by the spectrometer resolution. Hence, the term *x*(*Q*)*δ*(*ω*), *δ*(ω) being the Dirac delta function, accounts for either the elastic or perceived as elastic neutron-sample interactions and is, for example, often zero for bulk liquids at room temperature, in which diffusive motions dominate the QENS signal. *R*(*Q*, *ω*) is the instrument resolution, which, as depicted in Figure 3a, is convoluted to the experimental results and can be obtained by measuring the sample itself at temperatures as low as 2 K, a value easily reached using the available sample environment at large scale facilities, where the dynamic events usually cease. In other words, as discussed based on Figure 3a, this part of the equation depicts how the inherent characteristics of the instrument affect the shape of the detected QENS signal. *B*(*Q*, *ω*) is a background term that depends on both the spectrometer itself and the sample, as could also originate from the motions that are faster than the range of accessible energy transfer of the instrument (such as vibrational modes).

In the simplest cases, *S_incoh_*(*Q*, ω) can be described as a single Lorentzian function. For example, for a long-range translational motion in liquids, the single-particle self-correlation function, *G_self_*(*r*,*t*) must be a solution of Fick’s second law and is, therefore, a Gaussian function:

(5) $$\frac{\partial G_{s}\left(\vec{r},t\right)}{\partial t}=D\nabla^{2}G_{s}\left(\vec{r},t\right);\;G_{s}\left(\vec{r},t\right)={\left(\frac{1}{4\pi Dt}\right)}^{3/2}exp\left[\frac{-r^{2}}{4Dt}\right]\;\;$$

where *D* is the self-diffusion coefficient (that is, the diffusivity of the species with no chemical potential gradient). Consequently, *I_incoh_*(*Q*, *t*) is an exponential decay, $I\left(Q,t\right)=e^{-tDQ^{2}}$, and *S_incoh_*(*Q*, ω) assumes the Lorentzian form as:

(6) $$S_{incoh}\left(Q,\omega \right)=\;\frac{1}{\pi }\frac{\Gamma \left(Q\right)}{{\left(\Gamma \left(Q\right)\right)}^{2}+\omega^{2}}$$

where Γ(Q) is the half-width at half maximum (HWHM) of the Lorentzian QENS signal. In more complex systems, $S_{incoh}\left(Q,\omega \right)$ is often modeled as a sum of Lorentzian functions, accounting for fast and slow dynamics within the sample (hence broad and narrow components). In addition, $S_{incoh}\left(Q,\omega \right)$ may deviate from the Lorentzian shape and require alternative (and more complex) models for an accurate description. For example, a Cole–Cole distribution and a Fourier transformed stretched exponential function have been used in these cases, and both models bring along additional terms that reflect the heterogeneity of the chemical environment of the species under analysis [31,32,35].

Regardless of the model used to describe $S_{incoh}\left(Q,\omega \right)$, evaluating the *Q*-dependence Γ(Q) is a key step in the QENS data analysis and allows for extracting physical parameters that characterize the geometry and time scale of motions within the sample. In many cases, Γ(Q) can be described by a jump-diffusion model [37]:

(7) $$\Gamma \left(Q\right)=\frac{DQ^{2}}{1+DQ^{2}\tau_{0}}$$

where *τ*_0_ is the residence time between two diffusion jumps and *D* is the diffusion coefficient. In Equation (7), if *τ*_0_ = 0, one obtains the equation for a Fickian continuous diffusion. If *τ*_0_ ≠ 0, then one can also obtain, for systems with fairly low concentrations, a jump length, $L=\sqrt{6D\tau_{0}}$. In liquids, *L* often reflects the distance between two neighboring molecules. While Equation (7) satisfies all the scientific cases to be discussed in this review, other models (usually more complex) may eventually become necessary to accurately describe the evolution of Γ(Q).

Finally, if rotational motions play a relevant role in the QENS data, as discussed based on Figure 3d, one can often apply the model of isotropic rotational motion on a sphere, which considers reorientations of atoms in a molecule by small random angle changes. In such cases, *S_incoh_*(*Q*, ω) is also defined by a Lorentzian function (with a Q-independent Γ(Q)) but x(Q)≠0 in Equation (4) due to the presence of the EISF. In addition, one can explore another powerful feature of the QENS technique, since the Q-dependent behavior of the EISF provides valuable information about the geometry, and consequently the origin, of the motions under analysis. For example, the radii of the confined dynamics characteristic of some functional groups, such as methyl rotation, are well known and can be compared with parameters extracted by fitting the EISF using the appropriate models [38]. In addition, the EISF has been recently explored as a minimalist approach for interpreting the intermediate scattering function, *I*(*Q*, *t*). With this method, the QENS data are parameterized in terms of the EISF, a relaxation time scale, and the relaxation form, *τ*(*Q*) and *α*(*Q*), and subtle changes hidden in the spectra can be captured [39].

## 3. QENS and Cancer Cells

### 3.1. Experimental Considerations

**Figure 4:**
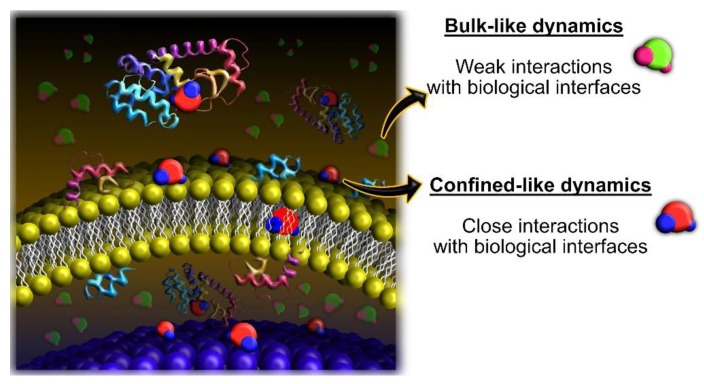
Illustration of the complex chemical environment within living cells and the different water populations. The green/pink molecules depict the bulk-like populations, which are subjected to weak interactions with the biological interfaces and are more abundant in cellular media (they are semi-transparent in the figure for presentation purposes). The red/blue molecules depict the confined-like populations, which are subjected to closer interactions with the biological interfaces and present features of confined dynamics that are not comparable with bulk water.

QENS has been used to probe micro-diffusion in living organisms for at least two decades. From the very beginning until the current days, many experiments have been performed with rather simple organisms, such as bacteria, which are valuable models to show that incoherent neutron scattering can provide useful (and interpretable) information on water dynamics within living systems [40,41,42,43]. The limits of the technique were gradually extended and, to this date, QENS allowed for investigating quite complex systems, including mammalian cells (including humans) and even multicellular organisms [44,45,46,47,48]. In all these cases, the very complex cellular environment poses a major challenge to describing the behavior and roles played by water. The richness of biological interfaces composed of membranes, proteins, and lipids within and around the cells promotes the distribution of water molecules as populations with distinct properties, as illustrated in Figure 4. Water molecules in weaker contact with biological interfaces may present bulk-like properties that are often comparable with pure water. Contrarily, as the biological interfaces impose soft confinement conditions, a fraction of the water molecules present confined-like dynamics.

**Figure 5:**
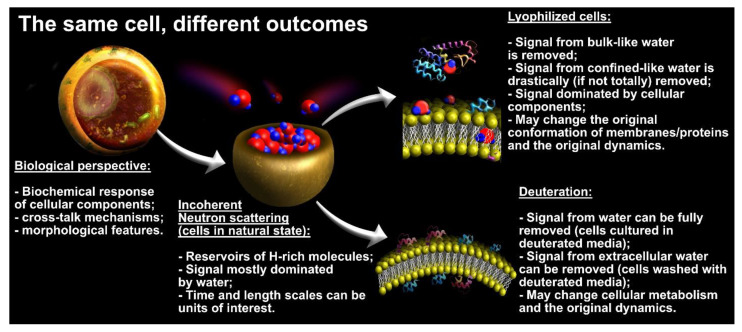
Schematics of the different outcomes obtained in QENS experiments with cancer cells.

For QENS experiments with cells and other living organisms, different approaches have been explored in the samples preparation to probe the dynamics of the distinct water populations. As represented in Figure 5, each approach requires compromises. If probed in their natural state, the living cells can be understood as reservoirs of (i) bulk-like and (ii) confined water populations and (iii) the several ^1^H-rich cellular components. On the one hand, with this methodology, one probes the natural dynamics of the system, while on the other, the QENS signal is often dominated by the bulk-like contributions, which hinders observation of the confined-like dynamics. It is common to resort to lyophilization procedures to remove the bulk-like populations of water from the cells as well as most of the confined molecules. With this, the dynamics from the remaining ^1^H-rich cellular components is highlighted, but the original structure of membranes and proteins can be disrupted (at least partially) [49,50] and the probed dynamics may not fully reflect the conditions within the cells in their natural state. Ultimately, one can also resort to exposing the cells to deuterated media. Culturing cells in a deuterated environment could be, in principle, a sound approach to drastically reduce the signal from cellular water (both confined and bulk-like) and highlight the relaxations from the remaining cellular components. Moreover, the data collected with such cells could be further subtracted from data collected with cells cultured in non-deuterated media, and one would be left with QENS signals mostly from the water itself. However, culturing cells in a deuterated environment has been shown to lead to considerable metabolic changes that could manifest as alterations in water dynamics [51]. Therefore, a less severe approach for deuteration has been used and consists of culturing the cells in non-deuterated media and sequentially washing these with deuterated saline solution [52,53,54]. By doing so, only the signal from extracellular water is drastically reduced in the QENS experiment.

### 3.2. What Have We Learned So Far with QENS and Cancer Cells?

**Figure 6:**
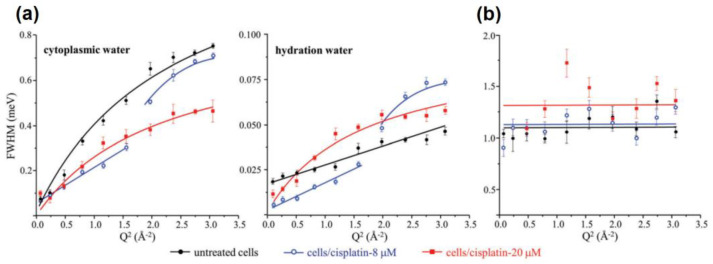
Variation of the full widths at half-maximum (FWHM) with Q^2^ for untreated and cisplatin-treated (8 and 20 mM) MDA-MB-231 cells in deuterated saline medium (washed), at 298 k: (**a**) Lorentzian functions representing the translational motions of intracellular water—cytoplasmic and hydration water; (**b**) Lorentzian function representing the internal localized motions within the cell. Reprinted with permission from ref. [53]. Copyright 2017 The Royal Society of Chemistry.

QENS experiments with cancer cells were pioneered by Marques et al. [53], who, in 2017, investigated the dynamics of intracellular water in the low prognosis human metastatic breast cancer cells MDA-MB-231. In this work, the cells were exposed to the anticancer drug cisplatin (cis-Pt(NH_3_)_2_Cl_2_), whose cytotoxic effect is mediated by DNA conformational rearrangements. The cells were cultivated in non-deuterated media, concentrated as pellets, and washed with deuterated phosphate-buffered saline (PBS) solution to remove the contributions from the extracellular water. Additionally, lyophilized samples were prepared to probe the dynamics of the cellular components with minimal interference from water. The QENS experiments were performed at the OSIRIS spectrometer (ISIS Pulsed Neutron Source of the Rutherford Appleton Laboratory, UK) with an energy resolution of 25.4 μeV (FWHM), which allows for the detection of motions on the order of 10^−10^ s and were combined with inelastic neutron scattering and optical vibrational spectroscopy. The spectra were collected at 298 K and fitted considering an elastic component, assigned to very slow motions from large cellular components and global motions of the macromolecules, and three Lorentzian-shaped contributions assigned to (i) the slow diffusion of water molecules in the hydration shells of biomolecules, (ii) the faster diffusion of non-hydrating water molecules, and (iii) the localized motions within the macromolecules and/or fast rotation of water molecules. Here, it should be noted that the choice of the model to fit any QENS data is hardly ever unique (e.g., in choosing between the model Q-dependence characteristic of Fickian vs. jump-diffusion vs. localized/rotational motion), and any chosen model could be, at least in theory, refined and improved. However, model-free comparison of the QENS data collected from different samples can always provide an unambiguous, even if more qualitative, indication of the effect of parameters of interest (e.g., drug concentration) on the microscopic dynamics under investigation. Interestingly, the authors reported that exposure to cisplatin induces distinct responses in the non-hydrating and hydrating water molecules. In the first case, the non-hydrating (bulk-like) water populations in cancer cells not exposed to the drug presented a diffusion coefficient, *D*, and residence time, τ_0_ (as defined in Equation (7)), of D = (1.04 ± 0.05) × 10^−9^ m^2^/s and τ_0_ = (1.0 ± 0.1) ps, which are on the same order of magnitude as expected for bulk water ($D_{H2O}$ = ~3 × 10^−9^ m^2^/s and τ_0-H2O_ = 1 ps). After exposure to cisplatin, the mobility of this water population is drastically damped and *D* drops to (0.19 ± 0.01) × 10^−9^ m^2^/s, whereas τ_0_ increases to (7.39 ± 1.16) ps (considering the highest dose of the drug used in the work, 20 mM). Meanwhile, the water populations confined in the hydration shells of biomolecules experience an increase in their mobility upon the action of cisplatin, with D changing from (0.0300 ± 0.0004) × 10^−9^ m^2^/s to (1.39 ± 0.13) × 10^−9^ m^2^/s. These trends are depicted in Figure 6, in which the bulk-like populations are defined by the authors as cytoplasmic water and the confined populations as hydration water. In the panels of Figure 6a, the full lines indicate the fitting of the QENS broadening with Equation (7). Here, another relevant feature depicted in the figure is that the treatment with the drug leads to distinct patterns in both the cytoplasmic and hydration water, in which the models used to fit the data at low Q-values, with *τ*_0_ = 0, do not describe the data at higher Q. As shown in Figure 6b, the component assigned to the localized motions within the macromolecules and/or fast rotation of water molecules only presents detectable differences between the cancer cells treated and not treated if the highest dose of the drug is used, although the lower dose already leads to changes in the components solely attributed to water dynamics.

Later on, in 2019, Marques et al. investigated the effects of cisplatin and Pd_2_Spm (Spm = spermine = H_2_N(CH_2_)_3_NH(CH_2_)_4_NH-(CH_2_)_3_NH_2_), whose cytotoxic effect is also based on targeting cellular DNA, in human osteosarcoma cells (MG-63) [55]. For this work, the cell pellets were also washed with deuterated PBS to remove the contributions from the extracellular water in the QENS experiments, which were performed at 310 K at the OSIRIS spectrometer and combined with Synchrotron-MicroFTIR. Like the previous work with breast cancer cells, the authors modeled the QENS spectra with an elastic component and three Lorentzian-shaped contributions. Following the same trend observed in the breast cancer cells, the mobility of the bulk-like water populations is reduced by the anticancer agents, and the opposite effect was observed in the confined dynamics. For the bulk-like water, *D* changes from (1.28 ± 0.01) × 10^−9^ m^2^/s in the untreated cells to (1.00 ± 0.01) × 10^−9^ m^2^/s after treatment with Pd_2_Spm and (0.89 ± 0.01) × 10^−9^ m^2^/s with cisplatin (considering the highest doses of the drugs used in the work). For the confined water, *D* changes from (0.17 ± 0.00) × 10^−9^ m^2^/s in the untreated cells (the null uncertainty is reported here as reported by the authors) to (0.72 ± 0.01) × 10^−9^ m^2^/s after treatment with Pd_2_Spm and (0.82 ± 0.01) × 10^−9^ m^2^/s with cisplatin (also considering the highest doses of the drugs used in the work). In this work, the authors rationalize that the increased mobility of the confined water molecules is associated with the biomolecules’ conformational rearrangement upon drug binding that leads to disruption of their highly structured hydration shell. In addition, the authors point out that the differences between the energies associated with the motions from confined and bulk-like populations were found to be less marked in the poorly metastatic osteosarcoma cells than in the highly metastatic breast cancer, and the impact of cisplatin on osteosarcoma’s bulk-like water is less pronounced than in the triple-negative breast cancer cells.

More recently, in 2020, Marques et al. used QENS to discuss the role of intracellular water in the normal-to-cancer transition in human cells [54]. For this, the group performed experiments with cancer cells and their non-tumorigenic counterparts. The following comparisons were conducted: triple-negative (metastatic) breast cancer (MDA-MB-231) vs. non-neoplastic mammary gland immortalized cells (MCF-12A) and androgen-independent prostate adenocarcinoma (PC-3) vs. normal prostate epithelium immortalized cells (PNT-2). Following the experimental procedures used by the same group in the above-cited works, the cells were washed with deuterated PBS, the QENS experiments were performed at the OSIRIS spectrometer at 310 K, and the spectra were modeled with an elastic component plus three Lorentzian-shaped contributions (confined- and bulk-like water populations and a Q-independent localized dynamics). In general, the bulk-like water populations were found to be more mobile in the cancer cells, whereas the confined molecules were less mobile. In this work, the most noteworthy difference between healthy and cancer cells is the difference in the fraction of elastic signals at 310 K. That is, in the cancer cells, a larger content of water molecules is engaged in motions detectable by the OSIRIS spectrometer as compared with the healthy ones.

In 2019, Martins et al. published a work in which QENS was combined with Inelastic Neutron Scattering and thermal analyses to evaluate the water dynamics in the breast cancer cell line MCF-7 before and after treatment with the anticancer drug paclitaxel [16]. Differently from the previous reports by Marques et. al, MCF-7 is considered to have low metastatic potential and paclitaxel does not target the cell’s DNA. Instead, this drug strongly interacts with the cellular membrane and promotes anticancer activity by inhibiting depolymerization of microtubules and causing mitotic arrest in cancer cells and ultimately apoptosis. In addition, the cells were not washed with deuterated media and were measured in their natural state. Therefore, the QENS signals were not devoid of information from extracellular water. For this work, the QENS experiments were performed at the BASIS spectrometer at the Spallation Neutron Source, USA, which provides an energy resolution of 3.5 μeV (FWHM) allowing for the detection of motions on the order of nanoseconds. Thermal analyses indicated that the action of paclitaxel increases the population of confined water within the cells and suggested an alteration in the structural organization of the water molecules. The latter result was confirmed by Inelastic Neutron Scattering, which also revealed that the vibrational modes attributed to proteins and DNAs were not altered by the drug. Hence, these results indicated that changes in the dynamics and structural organization of water could be detected even before changes in the dynamics from the remaining cellular components could be observed. A similar outcome was reported by the group when dielectric spectroscopy experiments were performed with the same cells before and after treatment with the same drug, paclitaxel [18]. Dynamic differences between the cells were clearly observed at very low temperatures when the global dynamics from the cellular components, and, consequently, the coupled motions from water molecules, ceased. Under this condition, single-particle dynamics from water molecules trapped within the frozen matrix cellular components could be exclusively probed.

**Figure 7:**
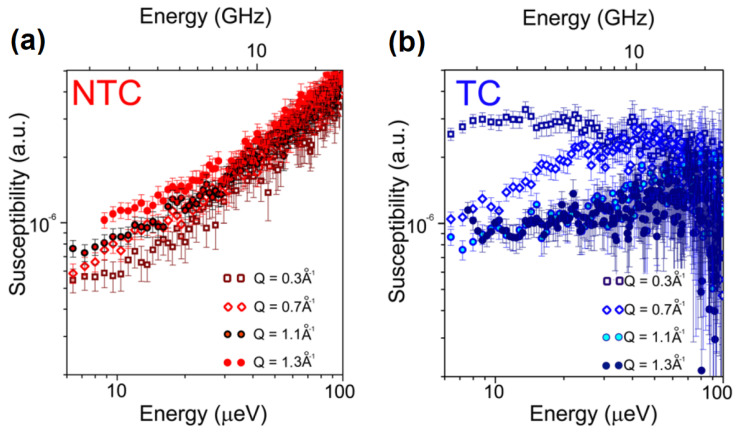
Dynamic susceptibilities obtained from QENS data for not-treated breast cancer cells (MCF-7), NTC (**a**), and breast cancer cells treated with 15nM of paclitaxel for 24 h, TC (**b**), reprinted from [16] available via the Creative Commons Attribution 4.0 International License (CCBY4.0, https://creativecommons.org/licenses/by/4.0/, 29 March 2022).

While differences between the cells treated and not treated with paclitaxel could be observed with dielectric spectroscopy at very low temperatures, QENS revealed a quite significant difference between these samples at room temperature, as shown in Figure 7. In the figure, the QENS data are presented as dynamic susceptibilities, obtained by $\frac{I\left(Q,E\right)}{n_{\mathrm{Bose}}\left(E\right)+1}$, where *I*(*Q*, *E*) is the intensity of the measured QENS signal, n_Bose_(*E*) = (exp(*E*/k_B_*T*) −1)^−1^ is the Bose population factor, and k_B_ is Boltzmann’s constant. In the dynamic susceptibility presentation, the characteristic diffusion/relaxation frequencies/times manifest themselves in the positions of susceptibility maxima. For the cells not exposed to the drug, the QENS signal was dominated by a very localized dynamics with a relaxation time of around 67 ps. After the action of the drug, a bulk-like non-localized dynamics was detected with *D* = (2.21 ± 0.11) × 10^−9^ m^2^/s. Finally, the authors raise another point of interest. The viability of the cells was tested after the QENS experiments and they found that around 70% of the cells (treated and not treated with paclitaxel) were still viable despite being exposed to non-optimal conditions for more than 12 h.

## 4. Perspectives for the Future

Despite its indispensable advantages as a probe of biological systems, QENS suffers from limitations such as a limited flux and a restricted (compared to many broadband probe techniques) dynamic range. Novel instrumentation is, therefore, one of the keys to expanding the application of neutron scattering to complex systems, including living cells. Currently, a new generation of backscattering spectrometers, including BASIS [56], DNA [57], and IN16B [58,59], is operational in spallation sources and reactor-based facilities and has already opened new doors to the high-resolution neutron spectroscopy/life sciences interface. As presented in this review, unraveling the complex dynamics within living organisms is gradually becoming routine in the QENS community, but, because of the flux limitations, long counting times are still required, and the samples are often exposed to non-optimal conditions for very long periods. Additionally, assessing different time and length scales in a single instrument is currently hardly possible, which prevents the elaboration of comprehensive dynamic maps for different cell lines, for example. In the following paragraphs, we present the perspectives brought by the two spectrometers to be built in the next years: MIRACLES, under construction at the European Spallation Source (Lund, Sweden), and BWAVES, which will be installed at the Spallation Neutron Source Second Target Station (Oak Ridge, TN, USA).

MIRACLES is envisioned as a quite versatile instrument in the research fields of life sciences and others and will benefit from an unprecedented flux, tuneable energy resolution in the range of 2–32 μeV, wide Q-range, flexible energy transfer with a range of ±0.6 meV around the elastic line, and an option of working in inelastic mode [60,61,62]. Finally, the time and space domain covered by Molecular Dynamics (MD) simulations is ideally matched to that offered by MIRACLES, which will allow for the description of complex systems thanks to theory/experiment combinations.

The other novel neutron scattering instrument, to be built at the Second Target Station of the Spallation Neutron Source, at the Oak Ridge National Laboratory in the United States, is the Broadband Wide Angle Velocity Selector spectrometer, BWAVES [63]. True to its name, BWAVES features a wide dynamic range of accessible energy transfers, from below 0.01 meV to above 500 meV. With a range of accessible energy transfers spanning 4.5 orders of magnitude in the measurable energy/relaxation time, BWAVES design makes possible, simultaneously, an extension of QENS spectrometry into the INS spectrometry range, and an improvement of INS vibrational spectrometry to include measurements of the slower relaxational motions. The former benefit, to QENS measurements, goes without further explanation, since the limited accessible dynamic range was always considered a disadvantage of QENS, especially when broad multi-component dynamics need to be analyzed. In this respect, BWAVES provides access to “unlimitedly” high, from the standpoint of QENS, energy transfers, when the QENS signal becomes superseded by the INS signal, thus enabling multi-component QENS signal analysis (from bulk-like water, hydration water, intracellular components). The latter benefit, to INS measurements, may be less intuitive, but, ultimately, could be of equal importance. As exemplified, e.g., by a study of dynamics of aqueous polyacrylic acid in dental restorative cements [64], as well as by our studies of breast cancer cells [16], measurements at several spectrometers, often at different facilities around the world, are often needed for sufficiently accurate characterization of both relaxational and vibrational dynamics in complex systems. As the complexity of the system under investigation increases, and especially with live systems, their evolution with time becomes increasingly probabilistic. For example, the state of two cellular cultures, designated for measurements at two different spectrometers, may diverge with time (due to different thermal histories, different sample holders, etc.), even if they originate from the same original culture initially split in half. Therefore, it is difficult to overestimate the capability of measuring the entire gamut of vibrational/relaxational dynamic processes using a single neutron spectrometer, as will be provided by BWAVES.

## 5. Final Remarks

In this paper, we review the works published to this date with a focus on the dynamics of water in cancer cells, as investigated with QENS. While promising, this is a bibliography under construction, and more references are needed to comprehend how far one can go in considering water as a potential target for treatment strategies or as a tool to understand the response of cancer cells under different circumstances and at different phases of the life cycle. Therefore, any investigations involving QENS and cancer cells, regardless of the cell line and sample preparation method, exposed to different stimuli, including drugs, radiation, environmental factors, heat, and others, greatly contribute to this topic.

Ideally, since living cells are very complex systems, we shall be able to, in the near future, divide the investigations not only into the different water populations but also into chunks of time scales. Then, the behavior of slow dynamics at the *ns* scale will be combined with the properties of motions occurring at the *ps* scale as well as with vibrational features. In the very end, we ought to understand how the microscopic dynamic events are related to those perceived on the time scale of days or even years, such as the development or remission of a tumor. With the next-generation neutron spectrometers, such as MIRACLES and BWAVES, sophisticated experiments will become possible in a fast timeframe, as different time scales will be accessible in single instruments.

Lastly, we ought to highlight the importance of combining QENS experiments with molecular dynamics simulations and other experimental techniques often more accessible to the general community. First, great improvements have been made on the simulations of water dynamics in cells, which will bring in a new dimension for the interpretation of QENS results and the detection of nuances often hidden in the experimental spectra. Ultimately, the QENS/molecular dynamics combination will provide us with the essential knowledge to interpret results obtained with more traditional and accessible techniques, such as thermal analysis, Raman, infrared spectroscopies, dielectric spectroscopies, and others.

## Data Availability

Data sharing not applicable to this article as no datasets were generated or analyzed in the current study.
